# Harvard Odontological Society

**Published:** 1903-02

**Authors:** 

**Affiliations:** Harvard Odontological Society


### HARVARD ODONTOLOGICAL SOCIETY.

*President Werner.*—Upon entering upon our twenty-fifth year as a Society it is perhaps well for a few moments to reflect how we were and how we are now. Beginning with five members, holding our first meeting on July 2, you see we have changed our birthday; we were really born in July, right after commencement day, after the ending of the school term, when there was a desire among those five members to come together and organize something which they had been, while at school, working among themselves as students. We have arrived now at our vigorous manhood of twenty-five years, with eighty-four, and over, active members.

It is well to remember that we confine our membership to graduates of the Harvard Dental School, and that is the first step in that direction of any dental society that we are aware of, outside of an alumni organization, a dental society which would meet monthly and confine itself to graduates of a certain school.

Then a very vigorous but cautious step we took when we adopted in our constitution and by-laws the requirement that each member should in his turn read a paper. That, in those years more than to-day, was a very decided step to take. It was the one thing that prevented our becoming a larger society in the early years. Men were afraid to belong to a society where they had to read a paper.

Then another unique thing that we adopted early, one of the first dental societies in the country, and certainly the first one in New England, and that is to meet in a hotel, at a banquet, at a dinner. Previous to that dentists used to meet in hired halls, hungry and tired, coming directly from their laboratories and from their chairs. They used to get angry, and quarrel and fight a good deal more than we do now. We found that a banquet, that the dinner-table, was a congenial and agreeable thing to all. So we were the first dental society to meet regularly at a banquet and have the meeting at a hotel.

We were the first dental society in New England that had a stenographer that took a minute report of everything said. That was quite a step.

It is well to consider these little marks in our foot-path during twenty-five years, and see what it has gradually led up to. We feel that no man should join this Society who is not willing to read a paper, and if he will not read a paper, the only thing for him to do is to get out, or never come in.

There has been a hinting in some directions already of limiting our members. Whether that is wise or not remains to be determined. When we began we certainly did not think of limiting members more than to graduates of the Harvard Dental School, and I have yet to see a reason why we should now limit them to any special number. I do not think our Harvard Dental School is going to limit its graduates. I do not think any department of the University is ever going to limit its graduates. I hope we shall never limit our membership. We want every good graduate of the Harvard Dental School to be a member of this Society; and when we have outgrown the quarters that this good hospitable hotel sometimes puts us into, a little room adjoining this that you are all so familiar with,—when we have outgrown that, we must come to a larger one like this; and when we have outgrown this, there are still some larger rooms. There is the Harvard Union, a club for Harvard men. You know about it: some of you are members, and some have been there, and some may not have been there. It is a very congenial place to go to. There is an atmosphere about it there that is nice. It seems that the time is coming when in that club every Harvard Society should meet and find accommmodations. I would like very much if the Executive Committee would consider that and would take us over there once, for an experiment, at least. We could save money, if that is an object. We would have a good comfortable room. Perhaps we would not have all the privileges of the club, because we are not all members, but we could have certain privileges; that could be arranged for.

I think we can honestly look forward to our twenty-fifth year as perhaps the best in our existence as yet. There is one other thing that I have been thinking of, and that is that we ought to have a committee of wide-awake young members to do something in the direction of original investigation. It would be taking a step that no other dental society, perhaps, has taken. Many of you younger men have been very faithful in attendance; you have been very patient listeners, some very mute, not having uttered a word. You all have shown good appetites. Some of you cannot be so very busy in your first years and could give some time to original investigation. I think our Society loses that element, and I should like to see a committee organized regularly every year who would stand for original investigation, and would make a report—a verbal or a written report—at every meeting of everything that is new in our whole dental literature. Some of us, perhaps many of us, do not read all the dental journals. Some of the younger men certainly have time, and read more than some of the older men. If they could condense all the practical points and report them at each meeting, it would give us a vast amount of material to think of and to discuss.

There is no absolute necessity of our adjourning at half-past eight or half-past nine. I remember the days when we used to adjourn at eleven and after. Our papers are short. We are not in a hurry; we are not hungry; let us come here and make the most of it. So I think that a committee of the kind I have mentioned, who would take original work and report on interesting things found in literature, or on practical points, would be a great addition to our written essays.

Without making further remarks, we all shall be glad to hear from Evan P. Wentworth, D.M.D., on "Winning the Confidence of Children." How essential it is to win the confidence of children no one will question who has been in practice. Many years ago on the Mediterranean there was born a man who stated some remarkable things about the simplicity of children,—that in order to get to the Kingdom you have to be born again and have to become as a little child. I think we could almost say that in order to succeed as dentists we have to be a lover of, and thereby win the confidence of, children. We shall be very glad to hear from Dr. Wentworth.

(For Dr. Wentworth's paper, see page 91.)

### DISCUSSION.

*President Werner.*—The subject is open for your consideration.

*Dr. Stanley.*—I have been very much interested in this paper. I think the subject is one of very great importance to every dentist, —winning the confidence of children. In order to do what is necessary to be done for the child you must first get the confidence of that child. Any way that that can be gained should be resorted to. I have always tried in my practice to get that confidence to start with. A patient comes to me once in a while where the pulp has to be destroyed in deciduous teeth. Never after an operation of that kind have I been able to have the absolute confidence of the patient.

The first operation should be a very short one, doing scarcely anything at all, but getting the attention on this or the other thing, anything to leave a pleasant impression on their minds the first time they visit your office.

I have in mind a family of three children, and they all had very poor teeth. One little fellow was one of those boys who will endure a good deal more than they will show: he was a plucky little chap. I think I had to commence on him when he was about three and one-half years old. He had a small mouth, and I had to fill all his back teeth, and it was no fun, either. I worked him along as carefully as I could. I soon had the confidence of those children, and now it stands me in good stead. They come right along and have their teeth attended to.

*President Werner.*—Many good thoughts must come out. I cannot help thinking of one. A few weeks ago a father informed me that Philip, a boy whose four lower incisors I had regulated in two months' time to quite a normal condition, had kept a little memorandum account of how many times I was pleasant and how many times I was cross. They are very cute observers. I have not found out how the balance stands. I intend to secure his confidence, as I have it now pretty thoroughly.

*Dr. Cooke.*—I was just wondering, while sitting here, how many of you gentlemen have children of your own. Certainly one of the best ways to gain the confidence of children is to practise it at home. And I have noticed, as a rule, that if a child does not behave in my office I have good reason to judge that it does not behave at home; and if it will not mind me and I cannot do anything with it, I come to the conclusion that its mother does not have any control over it.

One boy came in, and when I commenced to do anything for him he would yell. I said, "That is all right; you shout as loudly as you please." It was summer time and the windows were open, and he went at it, and I had to call him down. I said, "We will try a new tack." I sized him up, and I knew his aunt would stand by me. I said, "There are two ways to fill your teeth. They have got to be filled. There is an easy way and a hard way. There is the easy way; I will do it easily and gently; but if you shout, I am going right at it, and I shall not care where this instrument goes, but I am going right along with it." He said he could not keep still. I said, "You have got to. Now I am going to begin, and the minute you make a single bit of noise I shall cut in as hard as I please." I had the victory, and he never made a particle of noise.

I remember but one child that I was not able to work for: that was a boy four or five years of age, whom no one had ever been able to control. The parents would start for the opera, and he would shriek from the time they left until they got home. I never could do anything with him. He has always been on my mind, and I regretted my inability to conquer him. He has now grown up, and I have him again as a patient. He is not really a saint yet.

*Dr. Riley.*—I am afraid I cannot say much. I did not hear the paper, Mr. President, so I am hardly able to talk to the subject at all. But to relate experiences, I cannot have been many years in practice without having many experiences like Dr. Cooke's. One, that I think would tell the whole story, was a boy with much the record of Dr. Cooke's worst boy. He could not be got into the office. He was finally induced to enter the building by means of a ruse, and I was able to coax him into the chair. I remember, among many cavities that he had were two labial cavities in the centrals. The boy was eleven or twelve years of age. You can see the teeth were extremely neglected. I promised not to hurt him, and I had to keep that promise, but I did absolutely nothing the first day but to warm some gutta-percha and stick it in. The next day he came of his own volition, and I filled those two cavities properly, and five on the lower incisors. I won the boy's confidence, and he was a little man after that. I imagine he had been frightened.

*Dr. Cooke.*—I would like to ask Dr. Riley if he hypnotized the patient, or if he hypnotized the gutta-percha, so it would stay in a wet cavity.

*Dr. Riley.*—I dried the cavity partially.

*Dr. Cooke.*—I remember Dr. Riley was on record several years ago as placing gutta-percha in cavities that were not perfectly dry, and making it stay there. I never made gutta-percha stay where there was moisture.

*Dr. Naylor.*—It may be interesting for Dr. Cooke to attend one of Dr. Potter's lectures, where he makes gutta-percha stay on a glass filled with water.

*Dr. Cleveland.*—I only inject a remark. Dr. Meriam used to perform that experiment twenty years ago. He used to cover the surface with ether and resin and make it adhere to the inside of a tumbler filled with water. I have done it oftentimes, putting in temporary stoppings of gutta-percha and making it adhere to a moist cavity. I think if Dr. Cooke will try it some time he would succeed very readily.

*Dr. Wilson.*—Mr. President, naturally a man who has been in practice as long as I have has had experiences. I remember once a lady bringing in a little boy who needed some work done. The boy absolutely refused to open his mouth. He refused to stay in the chair. The mother asked me to force him to stay in the chair, and I rather objected; I did not like the idea of forcing the child. However, I did so. By the way, while I was carrying the child to the chair, somewhat forcibly, another patient saw me do it, and she said it was a horrid thing to do. If my memory serves me rightly, she went away and I never saw her again. I got the boy into the chair, and he was not one of the manly kind at all; he was simply stolid and ugly, and he would not open his mouth. Finally I had to force it open. Never, under any circumstances can you gain the confidence of a patient by so doing.

*Dr. Vaughan.*—I have tried a little different method. I have used bribery in a good many cases. I plan to have my children all come on Saturday and get it over with, and almost any Saturday I have two or three of them, and usually on that day you will see three or four children tagging me down to the drug-store for a soda, which I have promised if the patient will be good. I find it works quite nicely.

*Dr. Smith.*—Mr. Chairman, I think Dr. Wentworth has been very interesting. I was impressed with the gentleness which he outlined, and I also was doubly impressed with the gentleness of Dr. Cooke. His gentleness was of the strenuous kind.

I think one does not always fail because he has not children of his own, but you can adapt yourself to children's ways. If he has not children, he can love other men's children; and while I have no children of my own, I am very fond of children; I am interested in children; I am interested in what they do, and that goes a great way in mastering them.

Of course, children are made up of different temperaments, and you have got to meet the different temperaments the same as in grown-up people. There are times when you have to be very severe. In the majority of cases it is wise to be very gentle.

I was impressed with Dr. Wilson's relation of the case where he forced the child into the chair, and if I understood him correctly, he said he never won the confidence of that child, and that one would not be able to win the confidence of a child under such circumstances. I have had just the reverse experience in that kind of a case. The patient, a young lady now, is still a patient of mine. As a little girl, one of quite a large family of children, she ran the house, so to speak. She was brought to me, and she would not get into the chair. I had the nurse put the patient in the chair. After I got the child into the chair, I had to force her mouth open. I did so. I told her, to begin with, that I was not going to hurt her; that if I hurt her, if she would raise her hand I would stop instantly, trying to impress her with the fact that this operation was under her control. And I also use this term with children: I never speak of "cutting" a tooth, but I always use the term "scratching" a tooth. There is something about the word "cutting" with a child that I suppose causes blood to appear to them at once. It was an unfair battle, but of course it was necessary that she yield. I did not fill a tooth for her that day. She knew she was not hurt. She came after that, and I won her confidence, and it was not a great while until, when she came to her appointment, she would bring me flowers. She has remained a loyal patient ever since.

*Dr. Moffatt.*—There is one characteristic that has not been mentioned, and that is appealing to their pride, particularly in the case of boys. I had a patient about seven years old, who came with his mother. He had been once before and had got along very well, and I did not anticipate any trouble. But suddenly he began to cry and would not open his mouth. I stepped down and said I was not accustomed to working for that kind of a boy; that all my boys were good boys. I was sorry he did not have any more pride about it, but if he would come back again, I would be glad to have him. I kept on with what I had taken up, and in a few moments he came back. I appeared to be surprised to see him, but welcomed him. I had appealed to his pride.

*President Werner.*—All these that are brought out are good points, all gently hinting at what I wish to bring out, and that is truthfulness. You all know how essential it is, you who have had the experience, not to lie to a child; certainly not the first time. To-day I had a mother whose little boy had a left superior lateral extracted two days before, and she said, when she came in to-day, "Doctor, Roger thinks you are a very nice man." I said, "I am very glad." "You know last summer he had the tooth on the right side taken out up in the country, and the dentist told him he was not going to hurt him at all, just going to look at it; and then he concealed an instrument in his hand, and hurt him in pulling it. You did not tell him that, and he thinks you are a pretty nice man." One ought not to tell a child you will not hurt him, and then do it.

*Dr. Whitehead.*—There is one point that I thought of in regard to the stoical behavior of some children. I had one little patient who was very stoical. He was really a brave little fellow, and he would not give any sign of being tired or feeling any discomfort in the work that was being done for him. I think in such cases, in order to keep the confidence of such children, it is necessary to take that factor into consideration, and realize that we may sometimes keep a child too long, when he is really brave and able to bear it without signs of distress.

*Dr. Stoddard.*—I cannot really pose as a George Washington and say that I have never deceived any children in dental operations, but it has been my effort as much as possible to tell them the truth when I have had occasion to work for them. And when I have had occasion to take out any teeth for them I generally have told them I would give them ample warning when I was going to do it; I would not take them unawares and take out the tooth without their knowing it.

I question whether it is really our place to discipline other people's children; it is the parent's place to do that, and if the parents are not forceful enough to manage the child, I question whether it is our place as dentists to force the child to undergo a dental operation.

I believe in using kindness and fair dealing with children as much as possible, and try to persuade the child by all honorable means to have the operation performed, and not try to do too much at a time. I think very frequently as dentists we attempt too much at one time. You had better give a child a short sitting and put in one filling than to disgust him entirely with a number of fillings at one operation.

*Dr. Wilson.*—I have just happened to think of a case of a married woman whom I had as a patient at one time, who was an invalid for a number of years, and she owes all her ills and infirmities of the present time to her being forced to open her mouth in the dental chair and undergo a dental operation. She has a horror of this experience. She was forced into the chair and given ether.

*Dr. Smith.*—The case Dr. Wilson cites was a case, undoubtedly, where the patient was absolutely afraid; and I say to force a patient of that temperament to an operation would result seriously. It is the opposite case, where it is pure ugliness; forcing those people does not give them any trouble. It is a good thing for them.

*Dr. Clapp.*—I think this is a good time to remind you all of a little thing which you have probably forgotten, and that is that almost every temporary tooth that has to be extracted can be extracted without pain to the patient very easily, simply by the rapid-breathing process. I never had anything that has given so much comfort to little patients, where it is necessary to extract teeth, as this rapid breathing. I tell the child that if it will do just as I tell it, I will take out the tooth without hurting; and then I will show the child how to breathe. I tell them, "I want you to breathe rapidly and take good long breaths for half a minute, and then we will have the tooth out and you won't know anything about it." I tell them to take long breaths, breathing in and out easily and readily. After they have taken a dozen breaths like that, you can take out any temporary tooth and the child will not know it. I have even had to shake little patients to wake them up.

*Dr. Cooke.*—Have you ever had any bad results from the use of the rapid breathing?

*Dr. Clapp.*—No.

*Dr. Cooke.*—Do you ever try it in the excavation of sensitive cavities?

*Dr. Clapp.*—No; not to any great extent.

*Dr. Cooke.*—I have tried it with certain patients where the teeth were very sensitive,—grown up patients,—and there is no question but that it will help. Whether it is entirely the anæsthetic effect, or partly the taking the mind off the subject in hand and putting it on to the breathing, I do not know; but it does the work, I had a patient the other day that tried it, and she began to feel a tendency to faintness.

*President Werner.*—Are there any other remarks on this subject? If not, we will pass to our next, which is, "Crowns instead of Extensive Restoration with Gold." The discussion is to be opened, I think, by Dr. Clapp.

*Dr. Clapp.*—Some two months ago, I think it was, you will all remember that our president gave us a description of a very extensive restoration by means of filling. The time was late, or there were other things on the programme which required the time, and his case received no discussion. I said to one of the Executive Committee that it appeared to me that if that case should go without discussion it would leave the impression, particularly on the younger members of the Society, that these large restorations by means of filling with gold were usual and countenanced and recommended by the other members of the Society.

Now, I want it distinctly understood that in what I have to say I have no criticism, no adverse criticism, on this case that the president reported. But it does seem to me that the date of large restorations of gold filling have almost entirely passed. If I understood the report of that case properly, it was said that the patient believed that no crown could have been put on to his own teeth and have it clean and wholesome and durable. He had had one on and it had proved very unsatisfactory as regards wholesomeness and cleanliness. It looks to me just like this, that the gentleman who was ingenious enough to put a rubber dam on to that case and make a gold filling was ingenious enough to place a crown on to that tooth that would have been as perfect as a filling, and been neat and wholesome, with very much less discomfort to the patient and with as good, if not a better, chance of a successful operation as he had to making a successful operation by the means of the extensive gold filling.

As I said before, I think that the days of these very large gold filling restorations have passed. I do not wish at this time, and it is not the thing for me to do, to go into all the different kinds of crowns and caps that we have used that we consider pretty successful. There are many satisfactory crowns, many satisfactory ways of treating these teeth; but I will bring to your attention two or three cases that have been quite satisfactory, and these cases are in my own mouth.

In 1880, I think it was, I had the misfortune to break off my left superior first bicuspid. It was just about that time that the gold caps were beginning to be talked about. I made a simple straight gold ferrule for this bicuspid, and it was put on by a gentleman who was in the same building with me. That did perfect service, and if I remember rightly, was doing good service, except that the occlusion was not quite perfect, in 1889. It had been on nine years. I had that cap taken off, the end contoured, and the occlusion properly adjusted, and that cap was put back in 1890, and, so far as my recollection goes, it has never been disturbed from that time to this. It is in my mouth now, and is doing good service, and I should be glad to show it to you if you care to see it.

The second bicuspid on the same side was subsequently broken off, and a gold cap was put on to that in 1892. That did not last as well as the one I have just spoken of. In 1898, six years later, that cap loosened and came off. The pin that was put into the root (it was a cap with a pin put into the root) separated from the cap. It was not soldered to the cap. In 1898 this cap came off. The pin was again adjusted in the root and the cap put on, and is there now, doing good service.

In 1893 both of my right superior bicuspids gave out, and one of them was badly decayed and had been "tinkered" with more or less and a hole drilled through the side of the root, and it was in rather a weak condition. These teeth were treated in this manner (illustrates on the black-board). If possible, imagine that those are two bicuspid roots, quite badly decayed. I had put rather narrow bands of quite thin platinum around both of these teeth. These bands were put on as closely as they could be made to fit, and with a pressure, and were as tightly adjusted as they could be. Then there was a pin put down into the root and anchored, and then these bands were filled with amalgam. Both were treated in the same way. I was busy, and my friend who did the work was busy, and those were left. Of course those pins did not stick up very far, but they were good-sized pins, and they were not uncomfortable. I wore those bands without anything more being done to them for about a year. Then one of the bicusbids (I think it was the second one) was so very weak that the dentist who put the caps on for me said he thought it would be a good idea to join those two caps together, and so the crowns or caps were put on a sort of twin crown, just gold caps. They were put on in 1894, and are on to-day. That makes about eight years next July (I think it was in July).

I cite this as evidence that crowns can be put on, that they can be put in a cleanly condition, and that they will last a good long time. I often take a root and make a cap, the same as we all do, over the top and let the pin stick up a little like that, and cut this down so that, if it is a front tooth, or near the front, the gold will show very little, or none at all, and then, if it is a place where I do not wish to have the gold show, I have a porcelain crown cemented on.

Dr. Perry, of New York (I think), in some paper that he published some years ago, remarked that he liked to have the crown part the weakest part of the operation, so that if anything broke it would be the crown that would give out, leaving the cap and pin intact. If anything breaks, let the porcelain break, and then all you have to do is to have another top carved and put on. I think that is a very good point to have in mind when making crowns: have it so that if anything breaks, the top or crown part itself will give out before the anchorage. This same Dr. Perry published in the International Dental Journal of March, 1895, an article which is full of good things, and I want to read to you a short extract from it. Many of the young men have already heard this. (Reads extract.)

If any of you wish to see the specimens that are in my own mouth, I should be very happy to show them.

*President Werner.*—Those who wish to can look at the specimens, and at the same time we can listen to some other remarks on the subject. It is certainly a very interesting subject.

*Dr. Chase.*—I will say that I have had a number of cases in molars where the buccal mesio-distal walls were gone and very much of the palatal wall. The teeth have been devitalized, I have put posts in the root and with a matrix built those teeth down with amalgam. And I have some three cases that I know of where I contoured the teeth, and I have seen those fillings within the last year, and they have been in three years and are just as good as ever. I think they have given as good service as a crown.

*Dr. Stanley.*—Speaking about the restoration of molars reminds me of a case that recently came to me for treatment. The patient, a young man, had been suffering intermittently for two or three days, and located the pain in the right superior first bicuspid, which was an old offender, and, to use his expression, had been tinkered with a good deal. The tooth happened to have two roots. It required quite a little nursing before they could be permanently sealed, one treatment being to drill through the alveolus; but we finally succeeded and set a porcelain crown. Before the tooth was in a healthy condition the patient complained of pain in the lower jaw.

There was an innocent-looking gold cap on the first molar. He said that when he put his thumb-nail under the edge of the cap and lifted it up it relieved the pain. I lifted it up and off, and should think it might have given relief from the amount of pus I found. While living in Boston he had been in the hands of a Tremont Street operator.

The cap had been on but a few years, but the tooth had not been properly excavated and filled before this was done, so decay progressed and caused the death of the pulp. After the roots were in a healthy condition I filled them with paraffin and aristol, a preparation I frequently use. A root-canal dryer will cause this to flow readily as far as the canal has been opened. Then, after a thorough excavation, I treated the tooth with nitrate of silver. This I like to do when there is no possibility of it showing.

So much of the crown was gone in this case that I fitted a matrix band and built the tooth up with amalgam, the same as I did for Dr. Chase, and subsequently put a gold cap over this foundation. I feel as sure of the usefulness of this tooth for a long period of years as any of us can of a pulpless tooth. An upper molar on the left side was similarly treated.

The matrix is one of the most useful aids in filling many cavities. About the simplest form is the one I will pass around. You will observe it is a very thin piece of metal, one edge being folded upon itself, and holding the silk ligature, which you simply tie around the neck of the tooth.

If you will send one dollar to the International Dental Manufacturing Company, 503 Fifth Avenue, New York City, you can get several sheets of different thicknesses of very fine matrix metal, which I prefer to the flexible steel.

To-day I do not know what is a good crown. I use more of the Logan than any other. I am not satisfied with banding the root. I believe that in ninety per cent. of the cases the original idea of the band—viz., to prevent the root from splitting—is not only unnecessary, but harmful.

If the idea is adhered to, the strength required demands a band of some little width, the adjustment of which is very apt to sever the attachment of the gum. A ledge is left, irritation and inflammation follow, and in a few years receding of the gums. The normal condition should be preserved in all cases as nearly as possible.

*Dr. Boutwell.*—I would like to have some of the gentlemen who have had more experience in the matter than I, tell me what the difference is in the life of a tooth where it is built up with solid filling and a gold crown. It seems to me, in thinking it over, that possibly the thrust might be more evenly distributed over the surface of the tooth where it was built up with cement under these gold crowns, that the whole tooth would receive the thrust equally; whereas if a molar was built up with a large filling, one side would possibly receive a little more than the other. Is there any difference in the lasting qualities of the tooth?

*Dr. Reed.*—I was much interested in the doctor's question. I presume you mean as regards the permanency of the operation, the life of the tooth. It seems to me there could be hardly any question about the permanency of the operation. The gold crown would be far more permanent; and as regards the life of the tooth, I should say a well-fitted gold crown, in a case where there is very little of the crown left, would be the most permanent operation, particularly if the bite is very close.

Sometimes where a large mass of porcelain can be used, from an æsthetic point of view, an all-porcelain crown with a gold band is very much better, and perhaps fully as permanent, if the bite will allow it, because the strength of the bite of porcelain is in proportion to its bulk. If the bite is very close, most any sort of filling, or any sort of a porcelain crown would lack permanency, in my mind. It would certainly fracture and give out.

I think that bands and crowns can be constructed properly so that the articulation and interproximal spaces shall be about as they were before the decay commenced. Of course, it is hard to make rules about it, but if they are properly constructed, and we mix a little brains with the operation, we can have a very clean result and practically self-cleansing spaces, both mesial and distal.

The crown that Dr. Clapp spoke of I think is one of the best crowns, from an æsthetic point of view, that there is for anterior teeth. I remember it appealed to me when Dr. Smith spoke of it. I believe he described it. You have a transparency, and not the dead look that you get to a crown that is backed up with either gold or platinum. I think it is a very good crown.

*Dr. Estabrook.*—I would like to speak about treating the ends of roots with nitrate of silver. I think it is sometimes done indiscriminately, and results rather badly. I have seen cases where anterior teeth have been treated in that way, and even a little recession of the gums showed a jet-black root there with a nice crown protruding from it.

*Dr. Smith.*—Speaking of the Perry crown, I have had difficulty in getting them carved right. Of course, if a man like Dr. Dickinson does it, it is a different story. The old-fashioned pivot tooth was made with the hole going down in the centre. Consequently it was very weak. Now, in the Perry crown the difference is that the opening goes down directly towards the point, and you get a much stronger tooth.

In performing the operation of crowning a central incisor, you want as much depth of pin into the tooth as you can possibly get. As Dr. Clapp read from Dr. Perry, you would rather have the tooth give out than the root. It is rarely that I have a tooth give out. I have the hole made as deep as possible.

*Dr. Reed.*—I have had occasion several times to try to get the pin out of the porcelain after it had been cemented in. I boiled it in nitric acid for nearly half an hour, and had to give it up in the end. I had to break the porcelain in order to detach the pin. It is quite remarkable.

*Dr. Fernald.*—I have had the same trouble with teeth breaking off as Dr. Smith describes, and that leaves the posts so short that you cannot attach to them. In that case I carve the tooth myself, with a little box or platinum tube, quite heavy, fitting accurately over the end of the post, and the tooth cemented on, and I have had no trouble since.

*Dr. Dickinson.*—Before the subject is closed I would like to say that I have done a little carving, and have been in the habit of baking the pin into the crown, and I have asked some people I have carved for why they drilled the hole and then cemented the pin in, and they said it was easier to grind. I think stones can be had so that you can grind them perfectly well, and I think it is an easier way all round to have them baked right in, and then, even if your pin is shorter, it seems to be perfectly strong in the long run.

*President Werner.*—What is the objection to always baking them in?

*Dr. Dickinson.*—I do not know why it is done that way, unless they find it easier to be able to grind all over the surface of the crown instead of close to the pin.

*Dr. Chase.*—I have had a number of cases where I have used the Davis crown; in fact, I use that always in preference to others. You can bend the pin that comes in the Davis crown with a pair of pliers, and you can slip the crown on very easily, and it is much easier to grind up. You can bend it either way. You can bend the part of the pin that goes into the crown; it does not injure the pin in the least, and I find it is a great advantage.

*Dr. Smith.*—Why isn't that a factor in splitting the root?

*Dr. Chase.*—I never have had it happen.

*Dr. Dickinson.*—In answer to Dr. Chase, I should like to say that, if there is a case where the teeth tilt or tip in such a way that the crown with the pin baked or cemented in will not go directly into the root because of that bend, the pin can be bent so that it will be parallel to the line of the teeth before it is baked into the crown, and when the crown is baked you have a pin going in parallel with the teeth, which seems to clear away that trouble. Of course, there is no trace on the porcelain, because it is done before the porcelain is put on.

*Dr. Howe.*—I would like to ask Dr. Dickinson if he has used the mineral paints to match the other teeth? It occurred to me to-day to get some mineral paints, such as can be obtained at any art store, and use them to color teeth that you wish to put in to match the other teeth. You can get any shade you desire between the yellow and white, and I have tried it to-day on some artificial teeth, and it can be used to very great advantage, not only with crowns, but it seems to me that a person could color inlays to match perfectly. I think there would be no doubt as to its permanency. I would like to ask Dr. Dickinson if he has used them.

*Dr. Dickinson.*—The different shades are overcome by having the pure white, and gradations from that up to what I think is a deeper yellow than is ever seen in the mouth. Aside from that, you can make stain effects by the distribution of the enamel over the yellow,—by having it thick so that the yellow does not show through, or thin so that it does.

*President Werner.*—I wish Dr. Howe would explain to us precisely and minutely the direction in which he is experimenting and investigating in regard to these mineral paints that he uses, or anything else, on crowns that are more or less poorly matched. I understand he has quite a little experience in that direction.

*Dr. Howe.*—I must say that the idea is not my own at all: it is from Dr. A. H. Hart, who first suggested the mineral paints to imitate the tobacco stain. You not only can get the dark-brown stains, but any shade you wish. My experiments thus far have been only with these other shades. But it fuses at a lower temperature than the ordinary bodies, so that it can be used on almost any crown. I fuse it in an electric furnace. It takes about two minutes.

*Dr. Estabrook.*—I should think the objection to these mineral paints would be the same as the low-fusing porcelain bodies. In time they would change color in the mouth. Everybody that has had experience with very low-fusing bodies is aware that they change and darken in the mouth. I should think those mineral paints that have a large percentage of glass in them would change.

*Dr. Howe.*—These have not had the test of time, but it seems to me as if the stains that have been used for years upon chinaware, and have stood the test of wear, would not wear away in the mouth or change color in the least.

*Dr. Estabrook.*—I do not mean wear away, but change color. No doubt these glass fillings that have been put in years ago, if they had been left out of the mouth would not have changed color.

*Dr. Howe.*—I hardly think there would be any change with glass porcelain fillings. These mineral paints should have a background of porcelain, and the porcelain surely will be permanent, and your cement or background is not in the inlay.

*Dr. Stanley.*—Does Dr. Dickinson know anything about the crown material that White's people put on the market a short time ago?

*Dr. Dickinson.*—I never have used anything but the bodies that we make ourselves, so I do not know anything about that. Of course, the higher fusing a body is, the greater strength there is,—the less glass. Do you know whether those are high-fusing bodies?

*Dr. Stanley.*—It is not a particularly high-fusing body. I think it is lower than White's inlays.

*Dr. Dickinson.*—I should think the probabilities are it would not be as strong as Woodman's body.

*Dr. Stanley.*—Two cases of recent treatment come to my mind. One of my regular patients experienced some little trouble with the right lower second molar, such as might arise from a metal filling on the buccal surface. I removed such a filling, which had been in two years or so. The cavity was not deep, but very sensitive. I refilled it with copper cement. In a few days the patient came in and told me what a time she had been having. The face was somewhat swollen, though better than it had been. Every test I gave indicated live pulps in this and the adjacent teeth. Nature and a little time straightened the case out for me, and, so far as I know, the change of filling-material ultimately brought relief.

The other case was of a young man who received a fall from his wheel, which, with the aid of his pipe, broke the left lateral and cuspid, the latter being broken in a perfectly straight line across the labial surface, but extended under the gum on the lingual surface, about one-third of the tooth being broken off, with the rounded end of the pulp left well exposed. The lateral was not so badly broken. I gave him gas and removed the pulp. While debating what I had best do with the case he incidentally took the broken tip from his pocket. It went so accurately into position that I drilled into the cusp, selected a good strong iridio-platinum pin, and reset the broken point, with the result that it was the finest piece of "porcelain" work I had ever done.

*President Werner.*—Are there any specimens, or any incidents of office practice to be reported?

*Dr. Marvel.*—I was called out about a month ago to see a newly born baby, which was to me a very interesting case. Instruments had to be used at the birth, and the jaw was lop-sided. The left side of the jaw struck, and the right side was certainly half an inch from the upper ridge, to such an extent that the baby could not suck and there was danger of the child dying of malnutrition. I was called to see this child, and naturally I was somewhat feazed. I could see that a lead-pencil put between his teeth made it rather difficult for him to swallow. In consultation with the physician we decided to chloroform the child, and I took that little lower jaw and manipulated it. Of course it was not exactly gelatinous, but very flexible. I bent that jaw around so it came all right. When the baby came out it was naturally very hungry, and it took the breast readily, and the child has thrived ever since. The age when I saw the child was four days. Of course, the child does not take the breast until the third day anyway. On the extra day of course the child wanted food, and wanted it badly. Something had to be done. Whether this jaw was bent while being delivered or not, I do not know.

*Dr. Boardman.*—As this Society has increased very rapidly the last few years, in fact, doubled in the last seven or eight years, to meet the growth of the Society the constitution and by-laws need amending somewhat; and I would move you, sir, that a committee of three be appointed by the chair to alter or amend the constitution and by-laws and report the same at the next regular meeting for action. At the present time the secretary has to keep a record of all absentees, and it is a good deal of work, and not of much use. It may have been useful when the Society was small, but now it seems to me it should be amended in that respect, and in some others.

Voted.

On motion, adjourned.

H. W. Haley,
*Editor Harvard Odontological Society.*

